# Effect of Initial Rolling Temperature on Interfacial Reaction–Diffusion, Cladding Stability, and Tensile Failure of Industrially Hot-Rolled 316L/SWRH82B Clad Wire Rods

**DOI:** 10.3390/ma19132906

**Published:** 2026-07-07

**Authors:** Lei Zeng, Weiping Lu, Zhe Gou, Geng Zhou, Zecheng Zhuang, Xuehai Qian, Zhen Li, Jianping Tan

**Affiliations:** 1 College of Mechanical and Electrical Engineering, Central South University, Changsha 410083, China; 13698079602@163.com (L.Z.);; 2 State Key Laboratory of Precision Manufacturing for Extreme Service Performance, Central South University, Changsha 410083, China; 3 School of Mechanical and Electrical Engineering, Loudi Vocational and Technical College, Loudi 417000, China; 4 Technology Centre, Guangxi Liuzhou Iron and Steel Group Ltd., Liuzhou 545002, China; 5 Changsha WuJiang Intelligent Innovation Technology Co., Ltd., Changsha 410023, China

**Keywords:** clad wire rod, industrial hot rolling, 316L/SWRH82B, decarburization, carbon diffusion, EPMA, tensile stability, cladding thickness uniformity

## Abstract

To meet the combined requirements of high strength, intrinsic corrosion protection, and cost effectiveness for bridge cable wires, 316L/SWRH82B stainless-steel/high-carbon-steel clad wire rods were manufactured under industrial hot rolling conditions. Three initial rolling temperatures of 1000, 1024, and 1047 °C were investigated through metallographic observation, quantitative image analysis, EPMA characterization, SEM fractography, and tensile testing, with 15 specimens tested for each temperature group. The EPMA results, together with the metallographic observations, were used to evaluate carbon diffusion, interfacial elemental redistribution, and decarburization. As the initial rolling temperature increased from 1000 to 1024 and 1047 °C, the decarburized-layer thickness on the SWRH82B side increased from 7.42 ± 1.28 µm to 11.31 ± 1.74 µm and 18.15 ± 1.76 µm, respectively, whereas the carburization-affected-zone thickness on the 316L side increased from 48.36 ± 2.73 µm to 63.04 ± 3.06 µm and 68.73 ± 3.65 µm, respectively, demonstrating pronounced asymmetric interfacial reaction–diffusion. The average tensile strengths of the three groups were 1120.07, 1146.27, and 1152.28 MPa, with corresponding standard deviations of 14.83, 4.55, and 13.34 MPa and coefficients of variation of 1.32%, 0.40%, and 1.16%, respectively. Among the tested conditions, the 1024 °C group exhibited the lowest tensile-strength standard deviation and coefficient of variation, indicating the best tensile stability and mechanical consistency. Although the 1047 °C group achieved the highest average tensile strength, it also exhibited reduced cladding thickness uniformity and renewed mechanical scatter. All 45 tensile specimens were fractured on the SWRH82B side without obvious macroscopic interfacial delamination, indicating that the interface was not the preferential macroscopic fracture path under the present uniaxial tensile-loading condition. However, the intrinsic interfacial bonding strength was not directly quantified in this work. Therefore, 1024 °C is identified as the preferred initial rolling temperature for the specific billet geometry and industrial rolling conditions examined in this work, rather than a universally applicable value. The present study is limited to as-hot-rolled clad wire rods; corrosion performance, multi-pass cold drawability, and the final performance of bridge cable wires after drawing remain to be experimentally validated.

## 1. Introduction

Bridge cable wires are key load-bearing components in long-span bridges and are exposed throughout service to the combined effects of high tensile stress, chloride-containing environments, wet–dry cycling, fretting and fatigue loading. Previous studies have shown that corrosion pits, coating degradation, hydrogen-assisted cracking, stress corrosion cracking and corrosion fatigue can jointly reduce the residual strength, ductility and fatigue resistance of high-strength bridge wires [1,2,3,4,5,6,7]. Conventional protection systems, including hot-dip galvanizing, Zn-Al coatings, greasing, wrapping, outer sheathing and dehumidification, remain effective engineering measures for delaying corrosion. However, these strategies mainly provide external protection. Once local coating damage or sealing failure occurs, the high-strength steel substrate may still be exposed to aggressive environments, leading to localized corrosion and stress concentration. Therefore, introducing continuous metallic corrosion-resistant cladding into the wire itself provides a potential route for combining load-bearing capacity with intrinsic corrosion protection. In this study, SWRH82B high-carbon steel was selected as the core material because high-carbon pearlitic steels are widely used as precursors for high-strength steel wires, prestressing strands and bridge cable wires owing to their high strength potential and suitability for cold drawing [8,9,10]. Meanwhile, 316L stainless steel was selected as the outer cladding because its Cr-Ni-Mo alloying system provides good corrosion resistance and stable austenitic plasticity. Nevertheless, this material combination also introduces specific metallurgical risks: the high carbon content of SWRH82B increases the chemical driving force for carbon migration toward the stainless-steel cladding, which may induce substrate-side decarburization, cladding-side carburization, Cr-C interaction and near-interface property gradients during hot rolling.

Stainless-steel/carbon-steel clad products have been extensively investigated in the forms of clad plates, clad tubes, clad rebars, composite round steels and other bimetallic long products [11,12,13,14,15,16,17,18,19,20,21,22,23,24,25,26,27,28,29]. These studies have collectively shown that hot-roll bonding is not simply a physical contact process, but a coupled thermomechanical and metallurgical process involving oxide-film fragmentation, fresh-metal contact, interfacial shear, elemental interdiffusion, carbon migration, carbide precipitation, local recrystallization and heterogeneous deformation. For stainless-steel/carbon-steel clad plates and tubes, previous research has demonstrated that rolling reduction, heat treatment, substrate carbon content and interlayer design can strongly affect the decarburized zone, carburized zone, Cr/Ni diffusion, interfacial oxides, bonding strength and corrosion behavior [11,12,13,14,15,16,17,18,19,20,21,22,23,24,25,26,27,28,29]. Early and recent studies on hot-rolled stainless-steel/carbon-steel clad plates have further confirmed that carbon migration from the carbon-steel side can produce carbide precipitation and local hardening on the stainless-steel side, while chromium depletion or Cr-rich carbide formation may influence interfacial mechanical and corrosion-related behavior [14,15,16,23,24,28,29]. For bimetallic rods, composite round steels and clad rebars, recent studies have further emphasized the importance of deformation compatibility, profile evolution, interfacial bonding stability and geometric control during industrial forming and rolling [30,31,32,33,34,35,36,37]. In addition, studies on liquid–solid composite casting, localized interfacial mechanical response, bridge/building-oriented clad plates, heat-treatment regulation and surface-state control have further shown that the key concern in stainless-steel/carbon-steel composites has shifted from simply achieving metallurgical bonding to controlling the coupled microstructural, chemical, geometrical and property gradients formed after bonding [38,39,40,41,42].

However, several unresolved issues remain when these findings are extended to 316L/SWRH82B clad wire rods for bridge-cable-wire precursors. First, most existing studies focus on planar clad plates, clad tubes, clad rebars or larger composite round steels, whereas the present material possesses a closed annular cladding geometry. During industrial hot rolling, this geometry is subjected simultaneously to radial compression, circumferential metal flow, roll-groove confinement and interfacial shear. As a result, local deformation mismatch between the 316L cladding and the SWRH82B core may directly affect circumferential cladding-thickness uniformity. Second, compared with low- or medium-carbon substrates commonly used in stainless-steel/carbon-steel clad products, SWRH82B provides a much stronger carbon source. This may promote interfacial bonding through enhanced reaction–diffusion, but it may also aggravate carbon depletion in the pearlitic core, carbon enrichment in the 316L cladding and Cr-related carbide or short-range enrichment reactions near the interface. Third, unlike clad rebars that are often used directly after hot rolling, clad wire rods are intended as precursors for subsequent multi-pass cold drawing. Therefore, local cladding thinning, near-interface decarburization, carburization-affected zones, oxide/inclusion remnants and strength scatter generated during hot rolling may become important processing risks in later deformation. Since the present manuscript does not directly evaluate corrosion performance or cold drawability, these aspects are considered here as application-driven motivations and processing constraints rather than as validated outcomes.

On this basis, the central hypothesis of this study is that increasing the initial rolling temperature has a dual effect on 316L/SWRH82B clad wire rods. On the one hand, a higher temperature is expected to reduce deformation resistance, increase real interfacial contact, promote oxide-film fragmentation and enhance elemental interdiffusion, thereby improving metallurgical bonding. On the other hand, excessive temperature may intensify carbon migration from the SWRH82B core to the 316L cladding, accelerate substrate-side decarburization, strengthen cladding-side carburization/Cr-C interactions, increase circumferential cladding flow instability and enlarge batch-scale strength scatter. Therefore, the preferred initial rolling temperature should not be determined only by the highest average tensile strength, but by a comprehensive balance of interfacial bonding, reaction–diffusion moderation, cladding geometrical stability and mechanical consistency.

Accordingly, three actual industrial initial rolling temperatures, namely 1000, 1024 and 1047 °C, were selected in this study to investigate 316L/SWRH82B stainless-steel/high-carbon-steel clad wire rods manufactured under full-scale hot-rolling conditions. A metallographic observation, machine-vision-based quantitative measurement, EPMA elemental analysis, SEM fractography and statistical tensile evaluation were combined to clarify how initial rolling temperature governs interfacial reaction–diffusion, cladding-thickness stability, strength consistency and tensile fracture behavior. Through this approach, the present work aims to establish a coupled “temperature–diffusion–microstructure–geometry–fracture” framework and to identify the preferred initial rolling condition among the tested temperatures for industrial 316L/SWRH82B clad wire rods.

## 2. Materials and Methods

The present study investigates 316L austenitic stainless-steel/SWRH82B high-carbon-steel clad wire rods, in which the 316L stainless steel functions as a continuous corrosion-resistant outer cladding, while the SWRH82B high-carbon steel serves as the load-bearing core. The nominal chemical compositions of the two constituent materials are listed in Table 1. The high C content of SWRH82B provides the carbon source for interfacial carbon migration, whereas the Cr, Ni and Mo contents of 316L are closely related to corrosion resistance, austenite stability and the near-interface Cr–C interaction during hot rolling. The clad wire rods were fabricated through a composite billet route by sleeving a high-carbon-steel core rod with a stainless-steel tube, followed by heating, continuous hot rolling, coil laying and air cooling on the industrial production line of Liuzhou Iron and Steel Co., Ltd. (Liuzhou, China). Distinct from laboratory-scale hot-pressed composites, static diffusion couples or pilot-mill rolled specimens, all samples examined in this work were sectioned from finished wire rods produced under full-scale industrial hot-rolling conditions. Consequently, the subsequent analyses of interfacial reaction–diffusion, cladding geometrical stability and mechanical-property fluctuations are directly rooted in the thermal–mechanical history of real industrial processing.

### 2.1. Preparation of Composite Billets and Industrial Hot Rolling

**Figure 1:**
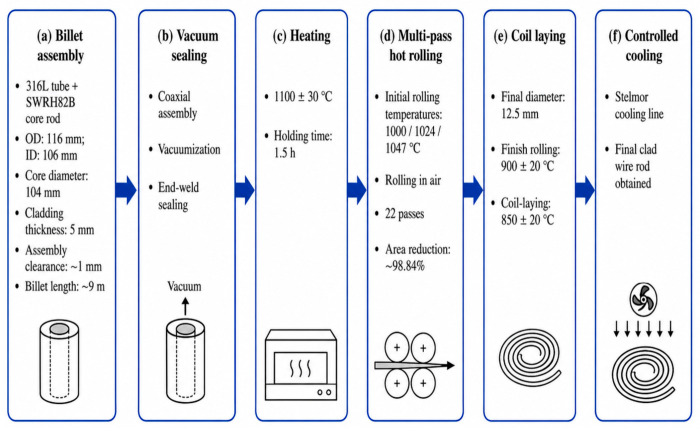
Schematic process flow of the industrial manufacturing route of the 316L/SWRH82B clad wire rod: (**a**) billet assembly; (**b**) vacuum sealing; (**c**) heating; (**d**) multi-pass hot rolling; (**e**) coil laying; and (**f**) controlled cooling.

The industrial manufacturing route of the 316L/SWRH82B clad wire rod is schematically illustrated in Figure 1, including billet assembly, vacuum sealing, heating, multi-pass hot rolling, coil laying and controlled cooling. First, the inner surface of the 316L stainless-steel tube and the outer surface of the SWRH82B core rod were subjected to clean-interface treatment to remove oxides, oil contaminants and other interfacial impurities. The two components were then coaxially assembled, vacuumized and sealed by end welding to obtain the composite billet for subsequent rolling, as shown in Figure 1a,b. The composite billet was then heated before being transferred to the industrial hot-rolling production line, as shown in Figure 1c,d. During rolling, coordinated plastic deformation was achieved under actual roll-groove confinement and continuous multi-pass rolling conditions. After rolling, the wire rod was coil-laid and subsequently cooled on the Stelmor cooling line, as shown in Figure 1e,f, thereby obtaining a finished clad wire rod consisting of a continuous 316L outer cladding and an SWRH82B core. The geometric parameters of the composite billet were specified to clarify the initial cladding/core configuration. The outer and inner diameters of the 316L stainless-steel tube were 116 mm and 106 mm, respectively, corresponding to an initial nominal cladding thickness of 5 mm. The diameter of the SWRH82B core rod was 104 mm after removing the oxide scale, leaving an initial radial assembly clearance of approximately 1 mm between the tube inner surface and the core rod. The billet length was approximately 9 m. Based on the cross-sectional areas of the 316L tube wall and the SWRH82B core rod, the initial cladding-to-core area ratio was approximately 0.205:1, corresponding to an initial cladding area fraction of approximately 17.03%. After industrial hot rolling, the nominal diameter of the finished clad wire rod was 12.5 mm. Based on the nominal billet outer diameter and the final wire-rod diameter, the cumulative cross-sectional area reduction was approximately 98.84%.

To evaluate the effect of initial rolling temperature on interfacial microstructure and property stability, three actual industrial initial rolling temperatures of 1000, 1024 and 1047 °C were selected as the processing variables. Before rolling, the composite billets were heated at 1100 ± 30 °C and held for 1.5 h. The rolling process was carried out in air on a full-scale industrial hot-rolling line. Each billet was continuously rolled through 22 passes and then subjected to controlled cooling on a Stelmor cooling line. For all groups, the finish rolling temperature was approximately 900 ± 20 °C, and the coil-laying temperature was approximately 850 ± 20 °C. Owing to the constraints of full-scale industrial production, the detailed pass-by-pass reduction, real-time rolling speed and complete cooling curve were not continuously recorded during the industrial trial.

### 2.2. Microstructural and Interfacial Elemental Characterization

**Figure 2:**
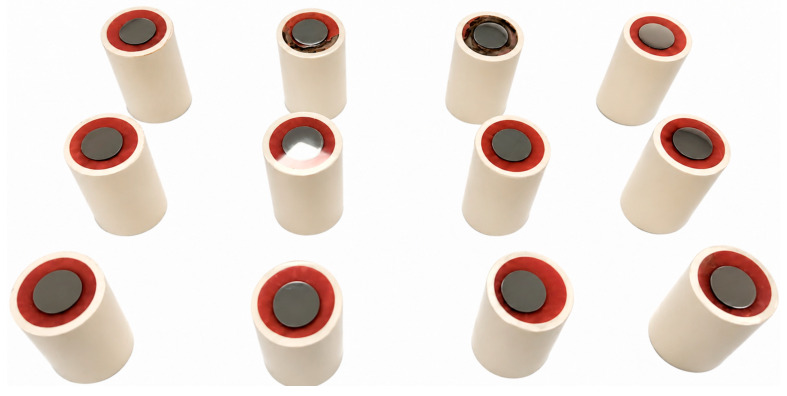
Representative cross-sectional metallographic specimens of the 316L/SWRH82B clad wire rod (The blank lines represent the resin and tube used during sample preparation).

Cross-sectional metallographic specimens were sectioned from the finished wire rods produced at different initial rolling temperatures, and representative specimens are shown in Figure 2. After cutting, mounting, mechanical grinding, polishing and final polishing, the two constituent materials were selectively etched. The SWRH82B high-carbon-steel side was etched using 4 vol.% nital to reveal the near-interface decarburized microstructure, whereas the 316L stainless-steel side was etched using an aqua-regia-based reagent to reveal the near-interface microstructure of the cladding and the carburization-affected zone.

Macro-metallographic examination and optical microscopy were employed to characterize the cladding profile, interfacial continuity, the decarburized region on the SWRH82B side and the carburized region on the 316L side. The thicknesses of the decarburized and carburized layers were quantitatively measured using a machine-vision-based method to improve the statistical comparability among different temperature groups. The machine-vision-based measurement procedure was further clarified in the revised manuscript. Briefly, each metallographic image was calibrated using its scale bar to determine the pixel-to-length conversion factor. The interfacial region of interest was cropped, converted to grayscale, and processed by contrast enhancement and median filtering. The boundaries of the decarburized layer and carburization-affected zone were identified by grayscale-threshold segmentation combined with local boundary-gradient recognition, followed by morphological opening and closing to reduce isolated noise and improve boundary continuity. The extracted boundaries were checked against the original metallographic images and manually validated to avoid artifacts caused by local etching contrast or edge effects. For each initial rolling temperature, six representative interfacial images/measurement groups were analyzed, with multiple local measurement lines taken in each image. The image-averaged value was used as one statistical measurement group, and the final results were reported as mean ± standard deviation, 95% confidence intervals and Min–Max values. In addition, EPMA line scanning and elemental mapping of C, O, Fe, Cr, Ni, Mn and Si were performed across the interfacial region to elucidate cross-interface elemental migration and local enrichment behavior. For each initial rolling temperature, six measurement groups were used for statistical analysis of the decarburized-layer and carburization-affected-zone thicknesses. The results are reported as the mean ± standard deviation, together with 95% confidence intervals and Min–Max values. The Min–Max values represent the overall thickness variation among different measurement groups, rather than measurement uncertainty alone.

### 2.3. Preparation of Tensile Specimens and Mechanical Performance Evaluation

**Figure 3:**
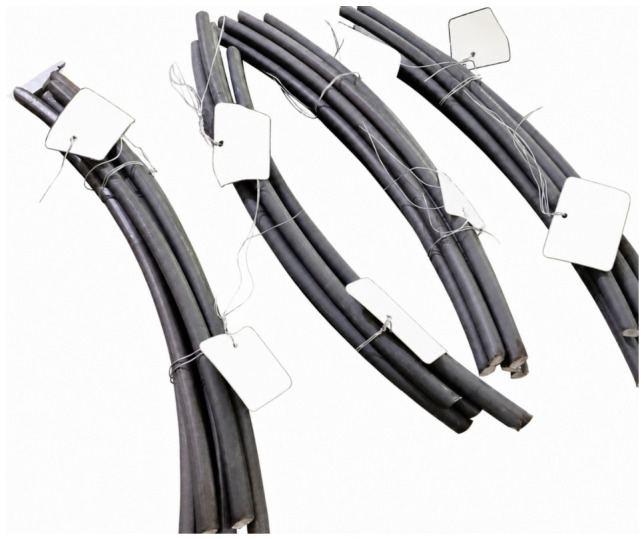
Representative tensile specimens of the 316L/SWRH82B clad wire rod with the original composite architecture retained.

To evaluate the mechanical properties and batch-scale stability of the clad wire rods produced at different initial rolling temperatures, room-temperature tensile tests were performed according to GB/T 228.1-2021 [43]. Fifteen specimens were tested for each temperature group. The specimens were prepared from the finished 12.5 mm clad wire rods, while the original 316L/SWRH82B composite architecture was retained without removing the cladding. Representative tensile specimens are shown in Figure 3. The initial specimen diameter was 12.5 mm, and a proportional gauge length of approximately 5d0 was used. The specimens were gripped using wedge-type grips and tested at room temperature on a SANS CHT4 series microcomputer-controlled hydraulic universal testing machine.

The tensile strength was calculated using the full composite cross-sectional area rather than the SWRH82B core area alone. The initial cross-sectional area was determined from the measured full diameter of each specimen; therefore, the reported tensile strength represents the overall load-bearing capacity of the as-hot-rolled clad wire rod. In this industrial batch test, ultimate tensile strength and reduction in area were recorded, whereas yield strength, total elongation, fracture strain, load-cell capacity and real-time crosshead-speed/strain-rate data were not systematically exported for all specimens.

After tensile testing, the tensile strength and fracture location of each specimen were recorded, and the average tensile strength and standard deviation were calculated for each temperature group. In this study, the stability of the industrial hot-rolling process was jointly evaluated in terms of average tensile strength, strength scatter and fracture-location distribution. Specifically, the average tensile strength was used to characterize the overall load-bearing capacity, the standard deviation was used to reflect batch-scale mechanical consistency, and the fracture location was used to determine whether failure was associated with interfacial bonding, cladding geometrical fluctuation or microstructural variation in the core. The tensile fracture surfaces and local failure features were examined by SEM. Because the tensile tests were conducted as part of an industrial batch evaluation, the specimen-level raw tensile curves and complete individual tensile records were not systematically exported in a form suitable for constructing reliable box-and-whisker plots. Therefore, only the statistically verified summary results, including sample number, mean value, standard deviation and coefficient of variation, are reported.

## 3. Results and Discussion

### 3.1. Cladding Geometrical Stability and Temperature Effect

**Figure 4:**
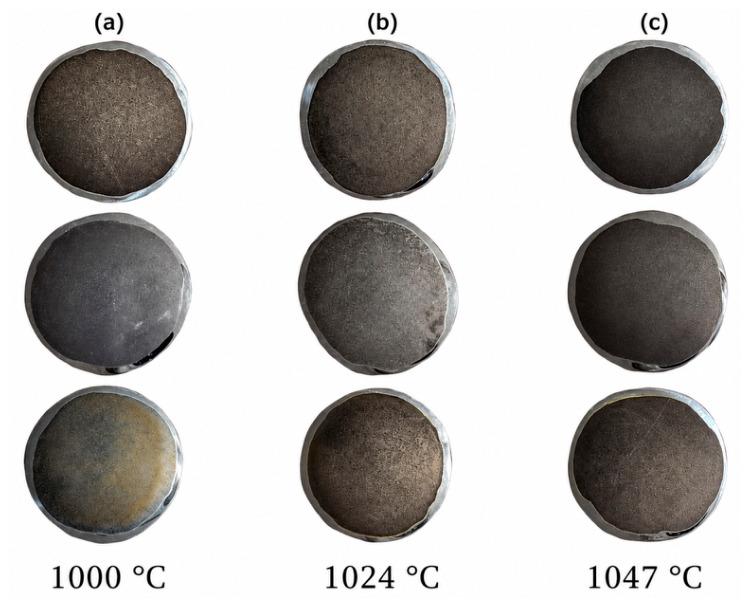
Enlarged macroscopic observations of the cladding profiles of the 316L/SWRH82B clad wire rods at initial rolling temperatures of (**a**) 1000 °C, (**b**) 1024 °C and (**c**) 1047 °C.

As shown in Figure 4, continuous outer claddings were formed in all 316L/SWRH82B clad wire rods rolled at initial rolling temperatures of 1000, 1024 and 1047 °C. No macroscopic cracks, opening defects or obvious interfacial delamination were observed in the cross-sections, indicating that the clean-interface billet assembly, vacuum sealing and industrial hot-rolling process effectively enabled stable bonding between the 316L outer cladding and the SWRH82B core. Nevertheless, the geometrical stability of the cladding exhibited a pronounced sensitivity to the initial rolling temperature. As the initial rolling temperature increased from 1000 to 1047 °C, the circumferential thickness difference in the cladding gradually increased, with the 1047 °C sample showing the most evident nonuniform flow between the thickest and thinnest regions.

To quantitatively evaluate circumferential cladding-thickness uniformity, representative cross-sectional images were analyzed for each initial rolling temperature, and the results are summarized in Table 2. The maximum thickness, minimum thickness, mean thickness, standard deviation, coefficient of variation, thickness difference and maximum-to-minimum thickness ratio were calculated. The coefficient of variation was defined as SD/Mean × 100%, and the thickness ratio was defined as Tmax/Tmin.

The 1024 °C specimen showed the lowest standard deviation, coefficient of variation and Tmax/Tmin ratio, indicating the best circumferential cladding-thickness stability among the tested temperatures. In contrast, the 1047 °C specimen exhibited the largest thickness difference and the highest coefficient of variation. Specifically, the thickness difference increased to 793.02 µm and the coefficient of variation reached 30.56% at 1047 °C, indicating a more pronounced circumferential thickness fluctuation. Although the Tmax/Tmin ratio of the 1000 °C specimen was relatively high because of a locally thin region, the 1047 °C specimen showed the largest absolute thickness difference and the highest coefficient of variation. Therefore, the overall statistical comparison indicates that excessive initial rolling temperature aggravated circumferential cladding-thickness fluctuation. At present, no specific industrial tolerance has been established for the circumferential cladding-thickness uniformity of as-hot-rolled 316L/SWRH82B clad wire rods, and the minimum residual cladding thickness required after subsequent multi-pass cold drawing has not yet been experimentally determined. Therefore, the present study does not define a strict pass/fail acceptance criterion. Instead, cladding-thickness uniformity is used as a comparative processing-stability indicator among the tested temperatures. A lower standard deviation, lower coefficient of variation, lower Tmax/Tmin ratio and larger minimum cladding thickness are considered more favorable for subsequent cold-drawing evaluation and cladding-integrity control.

This tendency is consistent with the “ear formation” and profile deviation reported by Li et al. [21] during hot rolling of clad rebars, where deformation incompatibility and circumferential redistribution of metal flow can occur between the outer stainless steel and the inner carbon steel. For the 316L/SWRH82B clad wire rod, increasing the initial rolling temperature can reduce deformation resistance and promote interfacial contact and metallurgical bonding; however, an excessively high temperature also enhances the local flowability of the 316L cladding under roll-groove confinement, thereby amplifying circumferential thickness nonuniformity. Unlike clad plates, in which cladding-thickness fluctuations mainly affect the local corrosion-resistance allowance, circumferential thickness nonuniformity in clad wire rods may be further transformed during subsequent multi-pass cold drawing into local stress concentration, variation in the residual protective cladding thickness and nonuniform interfacial shear strain. Therefore, cladding geometrical stability should not be regarded merely as a general appearance-quality issue, but rather as a key criterion for evaluating the industrial hot-rolling window and subsequent drawability of 316L/SWRH82B clad wire rods.

It should also be noted that circumferential cladding-thickness nonuniformity may not originate from temperature-driven flow mismatch alone. Billet eccentricity, radial assembly clearance, tube/core dimensional tolerance, local welding constraints and roll-groove alignment may also contribute to local thickness fluctuation. However, because the three groups were produced using the same billet-assembly route and the same industrial rolling line, the difference in increased thickness and coefficient of variation at 1047 °C suggests that excessive initial rolling temperature further amplified circumferential flow instability of the 316L cladding under roll-groove confinement.

### 3.2. Asymmetric Growth of the Substrate-Side Decarburized Layer and Cladding-Side Carburized Layer

**Figure 5:**
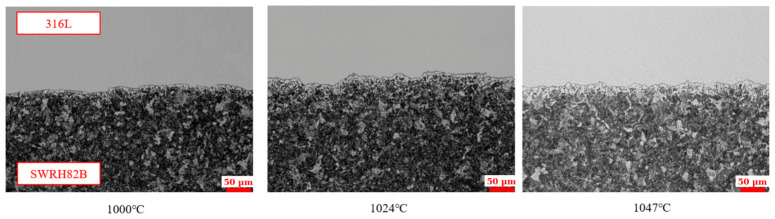
Metallographic images of the substrate-side decarburized layer in the SWRH82B core. The lighter near-interface band thickens with increasing initial rolling temperature.

**Figure 6:**
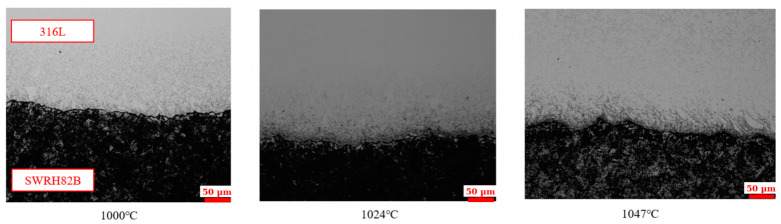
Metallographic images of the cladding-side carburization-affected zone in the 316L cladding. The near-interface affected zone is related to carbon migration and Cr–C interaction.

**Figure 7:**
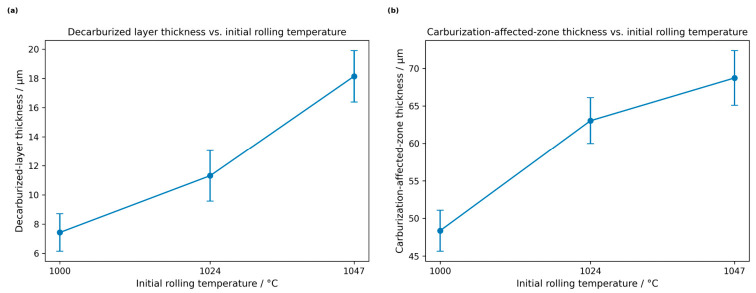
Error-bar plots of reaction–diffusion layer thickness versus initial rolling temperature: (**a**) decarburized layer on the SWRH82B side and (**b**) carburization-affected zone on the 316L side. Error bars represent standard deviation (n = 6).

Near-interface metallographic observations, as shown in Figure 5 and Figure 6, reveal that distinct composition–microstructure gradient zones formed on both sides of the 316L/SWRH82B composite interface. However, the diffusion layers on the two sides were not mirror-symmetrically distributed, but instead exhibited pronounced asymmetric growth. The machine-vision-based quantitative results are summarized in Table 3 and Table 4 in the form of mean ± standard deviation, and their temperature-dependent evolution is further shown in the error-bar plots in Figure 7. As the initial rolling temperature increased from 1000 °C to 1024 °C and 1047 °C, the decarburized-layer thickness on the SWRH82B side increased from 7.42 ± 1.28 µm to 11.31 ± 1.74 µm and 18.15 ± 1.76 µm, respectively. Correspondingly, the carburization-affected-zone thickness on the 316L side increased from 48.36 ± 2.73 µm to 63.04 ± 3.06 µm and 68.73 ± 3.65 µm, respectively. The Min–Max values in Table 3 and Table 4 represent the overall measured thickness variation among different measurement groups. These statistical results confirm that both reaction–diffusion regions thickened with increasing initial rolling temperature, while the 316L-side carburization-affected zone remained much thicker than the SWRH82B-side decarburized layer. The carburized layer on the cladding side was consistently much thicker than the decarburized layer on the substrate side, indicating that carbon migration from the high-carbon SWRH82B side to the 316L side did not simply produce a symmetric concentration gradient across the interface. Instead, it induced microstructural responses and etching-contrast variations over a wider region on the 316L side. More notably, when the initial rolling temperature was further increased from 1024 °C to 1047 °C, the growth of the carburized layer became markedly slower, whereas the thickening of the decarburized layer accelerated substantially. This phenomenon, characterized by the larger absolute thickness of the carburized layer but a faster high-temperature growth rate for the decarburized layer, indicates that the 316L/SWRH82B interface is not a binary diffusion couple governed solely by single Fickian diffusion. Rather, it should be regarded as a coupled reaction–diffusion layer jointly affected by carbon migration, possible Cr–C interactions or short-range elemental enrichment, austenite stability, pearlite decomposition and hot-rolling-induced plastic deformation.

The temperature-dependent evolution of the reaction–diffusion layers is further shown in Figure 7 with error bars representing the standard deviation of six measurement groups. The decarburized-layer thickness on the SWRH82B side increased continuously with temperature, especially between 1024 °C and 1047 °C. By contrast, the carburization-affected zone on the 316L side showed a slower growth tendency in the higher-temperature interval. These results support the asymmetric and nonlinear growth behavior of the two reaction–diffusion layers.

From the perspective of reaction–diffusion coupling, the asymmetric growth of the decarburized layer and carburized layer on the two sides of the 316L/SWRH82B interface mainly originates from the large carbon-content difference between the high-carbon SWRH82B core and the 316L cladding. Compared with commonly used substrates for clad rebars, such as HRB400E and 20MnSiV, SWRH82B possesses a higher carbon-source intensity, making it more prone to generating a cross-interface carbon flux from the substrate side toward the 316L side during hot rolling. After carbon depletion, the stability of cementite in the near-interface pearlite of SWRH82B decreases, and the local microstructure evolves toward ferrite or low-carbon transformed structures, thereby forming a decarburized layer. In contrast, carbon entering the 316L side does not diffuse solely in the form of solid solution; it can also be trapped by Cr, inducing Cr-enriched carbides, short-range enriched regions or etching-contrast variations, which result in a carburization-affected zone on the cladding side that is much thicker than the decarburized layer on the substrate side. As the initial rolling temperature increases, the carbon diffusion coefficient, real interfacial contact area and fast diffusion pathways introduced by hot-rolling deformation, such as dislocations, grain boundaries and fragmented oxide films, increase simultaneously, causing the interfacial evolution to deviate markedly from a static one-dimensional Fickian diffusion process. At 1000 °C, interfacial diffusion and reaction are relatively limited; at 1024 °C, carbon migration, Cr trapping and metallurgical bonding reach a more coordinated state; by contrast, at 1047 °C, carbon out-diffusion from the substrate side is significantly intensified, pearlite decarburization is aggravated, and the decarburized layer thickens rapidly, while the growth of the carburized layer on the 316L side tends to slow down. This indicates that the interface enters a stage characterized by accelerated substrate-side decarburization and nonlinear reaction–diffusion growth on the cladding side. This phenomenon is mechanistically consistent with the decarburized and composite regions observed by Li et al. [20] in clean-interface vacuum hot-rolled clad rebars, the conclusion of Liang et al. [24] that the carburized austenitic region is highly sensitive to the intermediate and finish rolling stages, and the observation of Chen et al. [11] that carbon concentration differences influence the manifestation of decarburized/carburized layers in 304/carbon-steel clad plates. However, because a high-carbon SWRH82B core is used in the present study, the interfacial carbon chemical-potential difference is substantially amplified, giving the 316L/SWRH82B system a stronger driving force for carbon migration and a more typical asymmetric reaction–diffusion behavior. Therefore, the decarburized and carburized layers observed here are not a simple extension of those reported for clad rebars or clad plates, but are rather an intensified diffusion–reaction behavior arising from the combined effects of the high-carbon core, the high-Cr 316L cladding and industrial roll-groove-constrained hot rolling.

Such asymmetric growth of the diffusion layers has a direct impact on the subsequent processing and failure behavior of the clad wire rod. On the one hand, moderate elemental interdiffusion helps eliminate abrupt chemical discontinuities across the interface and improves metallurgical continuity, which is one of the main reasons why no macroscopic interfacial delamination occurred in any of the three sample groups. On the other hand, excessive decarburization weakens the pearlitic strengthening capacity near the SWRH82B interface, forming a locally softened banded region, whereas carburization and Cr-related precipitation on the 316L side may increase near-interface hardness and enhance local embrittlement or corrosion susceptibility. Together, these effects generate an asymmetric property gradient: the interface itself is no longer the weakest region, but the softened zone, hardened/precipitation-affected zone and geometrically nonuniform region on either side of the interface may become new failure sources during subsequent tensile loading or cold drawing. In particular, under the 1047 °C condition, the rapid thickening of the decarburized layer indicates a significantly increased risk of near-interface softening on the substrate side. Even if the average tensile strength is slightly increased, this may be achieved at the expense of batch-scale stability and subsequent drawability. Therefore, the asymmetric growth of the substrate-side decarburized layer and the cladding-side carburized layer provides key microstructural evidence for understanding the transition in 316L/SWRH82B clad wire rods from “a bonded interface” to “a shifted system-level weakness”.

### 3.3. Carbon Migration, Possible Cr–C Interaction and Local O/Mn/Si Enrichment

**Figure 8:**
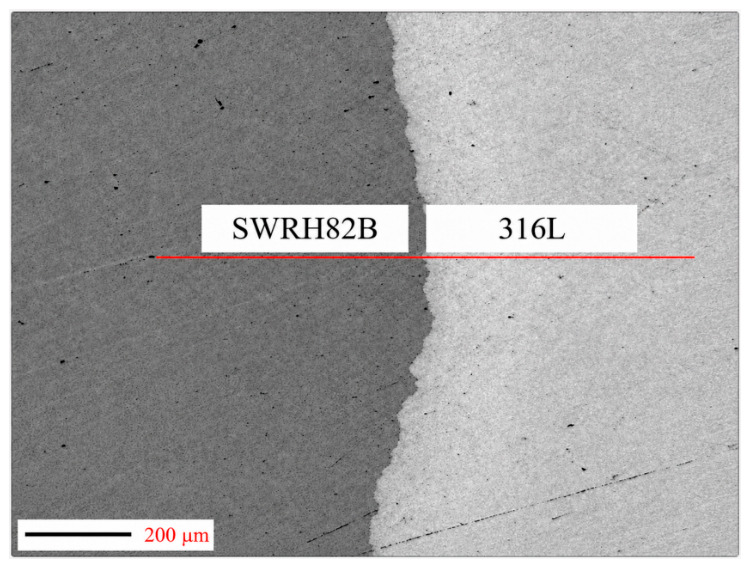
EPMA line-scan position. The red line indicates the EPMA detection position.

**Figure 9:**
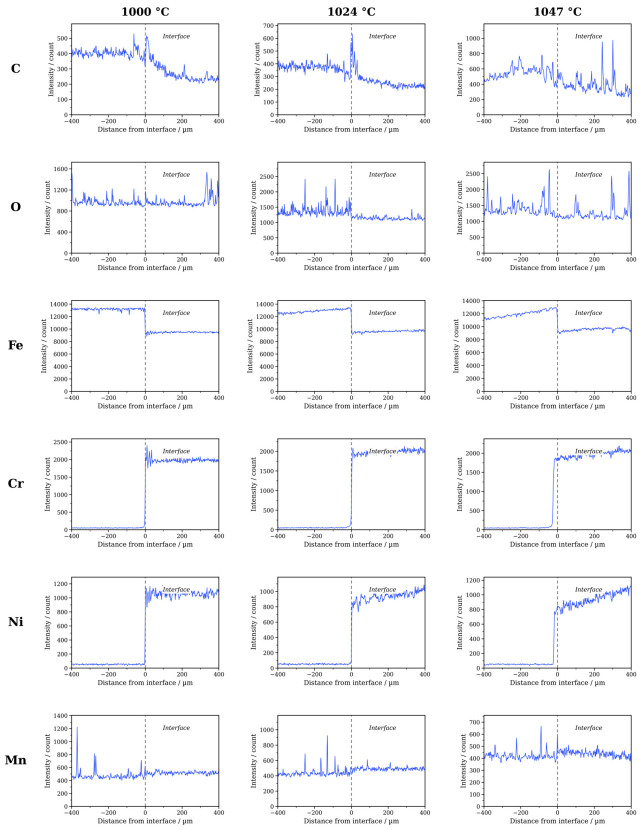
EPMA line-scan results across the 316L/SWRH82B composite interface at initial rolling temperatures of 1000 °C, 1024 °C and 1047 °C. The x-axis represents the distance from the interface in µm, and the y-axis represents elemental intensity counts. The vertical dashed line in each panel marks the interface position. Negative distances correspond to the SWRH82B side, whereas positive distances correspond to the 316L side.

As shown in Figure 8, the EPMA line-scan path was arranged perpendicular to the composite interface. The red line starts from the SWRH82B high-carbon-steel core, traverses the 316L/SWRH82B composite interface and extends into the 316L stainless-steel cladding. This line-scan path covers the near-interface decarburized region on the SWRH82B side, the interfacial transition zone and the affected region on the 316L side, thereby reflecting the redistribution of principal elements across the interface. EPMA line scans were performed for the 1000 °C, 1024 °C and 1047 °C specimens, and the corresponding results are shown in Figure 9.

A local near-interface carbon enrichment was observed on the 316L side. To avoid implying a thermodynamically verified uphill-diffusion mechanism, this feature is described here as apparent near-interface carbon enrichment associated with carbon migration from the SWRH82B high-carbon-steel core into the 316L cladding. Based on the concentration transition regions in the line-scan profiles, the approximate effective affected distances of C on the 316L side were about 50 µm, 65 µm and 70 µm for the 1000 °C, 1024 °C and 1047 °C samples, respectively. The corresponding effective transition distances of Cr near the interface were approximately 12 µm, 18 µm and 25 µm, respectively. These results indicate that increasing the initial rolling temperature promoted interfacial elemental redistribution and enlarged the reaction–diffusion-affected region, especially for carbon redistribution on the 316L side.

Since direct phase-identification evidence, such as TEM, XRD, EBSD phase mapping or high-resolution SEM-EDS particle analysis, was not obtained in the present work, Cr trapping, Cr-enriched carbide formation and precipitation are not claimed as directly confirmed phases. Instead, the local redistribution of C and Cr is discussed more conservatively as possible Cr–C interactions or short-range elemental enrichment near the interface.

Meanwhile, the EPMA line-scan results also show local enrichment of O, Mn and Si near the interface. This feature may be associated with oxide-film fragments, oxide particles or complex inclusion remnants introduced during billet assembly and hot rolling. However, because particle-level point analysis and backscattered-electron imaging were not performed in this work, the exact phase identity and morphology of these O/Mn/Si-rich regions cannot be determined. Therefore, these features are discussed as possible oxide- or inclusion-related remnants rather than as directly identified oxide particles. Within the present industrial hot-rolling window, the main issue at the 316L/SWRH82B interface is therefore not only whether bonding can be achieved, but also whether local elemental redistribution and possible oxide/inclusion-related remnants may affect subsequent cold drawing stability and service reliability.

**Figure 10:**
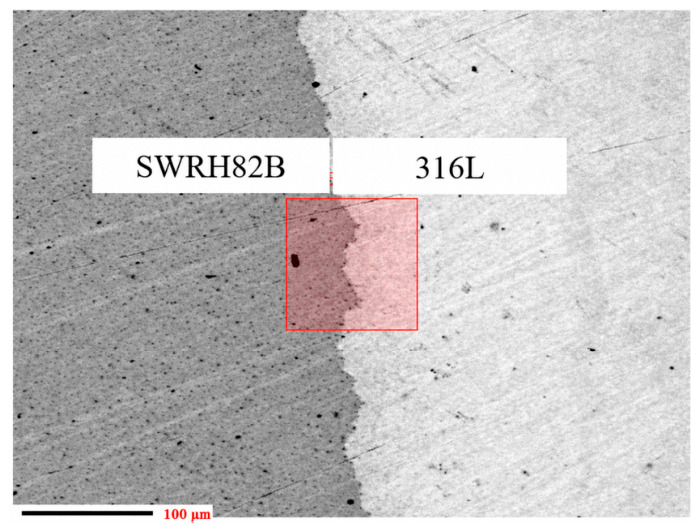
EPMA mapping area of the representative near-interface region in the 1047 °C specimen. The red square indicates the region selected for EPMA detection.

**Figure 11:**
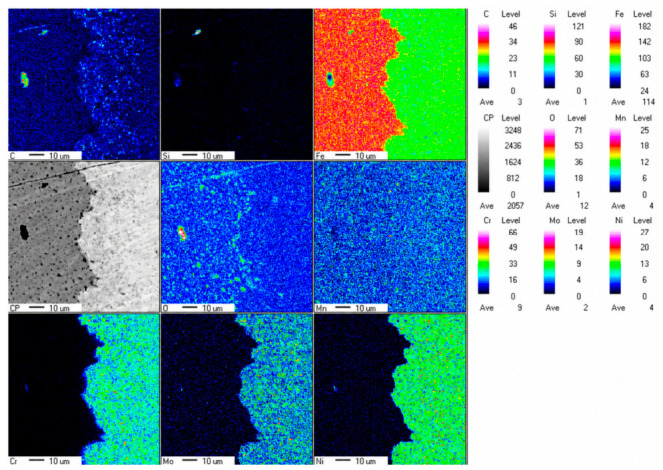
Representative EPMA elemental mapping results near the 316L/SWRH82B composite interface of the 1047 °C specimen.

To further illustrate the two-dimensional elemental distribution near the 316L/SWRH82B composite interface, representative EPMA elemental mapping was performed on a near-interface region of the 1047 °C specimen, as shown in Figure 10. The selected mapping area spans the SWRH82B substrate, the composite interface and the 316L cladding, thereby showing the local spatial distribution of C, O, Fe, Cr, Ni, Mn and Si near the interface. The corresponding EPMA mapping results are presented in Figure 11.

The elemental mapping results are generally consistent with the line-scan profiles and show local enrichment of C on the 316L side near the interface. This result supports the interpretation that carbon migrated from the SWRH82B high-carbon-steel core toward the 316L cladding and produced local near-interface carbon enrichment. Local O, Mn and Si enrichment was also observed near the interface, which may be related to oxide- or inclusion-related remnants. However, because the mapping result was obtained from a representative near-interface region rather than from all temperature groups, it is used here only to illustrate the local two-dimensional elemental distribution. The temperature-dependent comparison of interfacial elemental redistribution is mainly based on the EPMA line-scan results obtained from all three temperature groups.

### 3.4. Tensile Properties, Statistical Stability, and Transition of Fracture Location

The tensile strengths of the three groups of specimens processed at different initial rolling temperatures were tested, and the results are summarized in Table 5. The average tensile strengths were 1120.07 MPa, 1146.27 MPa, and 1152.28 MPa, respectively, indicating an overall increasing trend with increasing initial rolling temperature. However, considering only the average strength would obscure the most critical industrial information. The standard deviations of the 1000 °C, 1024 °C, and 1047 °C groups were 14.83, 4.55, and 13.34 MPa, respectively, corresponding to coefficients of variation of approximately 1.32%, 0.40%, and 1.16%. These results demonstrate that the 1024 °C condition not only achieved a relatively high strength level but also provided the most stable batch-to-batch consistency. Although the 1047 °C condition slightly increased the average tensile strength, its statistical dispersion increased markedly. For a precursor wire rod intended for bridge cable wires, the latter condition should therefore not be regarded as a superior processing route, but rather as a “strength-enhancing temperature” instead of a “stability-optimizing temperature”. In addition to tensile strength, the average reduction in area was added to Table 5 as a ductility-related indicator. The average reductions in area values of the 1000 °C, 1024 °C and 1047 °C specimens were 36.1%, 38.9% and 36.9%, respectively. Among the three groups, the 1024 °C specimen exhibited the highest reduction in area, together with the lowest tensile-strength standard deviation. This indicates that the 1024 °C condition provided a better balance between strength consistency and plastic deformation capacity. In contrast, although the 1047 °C specimen showed the highest average tensile strength, its reduction in area decreased compared with that of the 1024 °C specimen, suggesting that excessive initial rolling temperature did not further improve ductility. Therefore, the preferred rolling condition should be evaluated by considering both strength stability and ductility-related behavior. In addition, the fracture locations of all tensile specimens were recorded after testing. All 45 specimens, including 15 specimens for each initial rolling temperature, were fractured on the SWRH82B side. No specimen fractured along the 316L/SWRH82B interface or within the 316L cladding. This result indicates that, under the present uniaxial tensile-loading condition, the composite interface was not observed as the preferential macroscopic fracture path.

**Figure 12:**
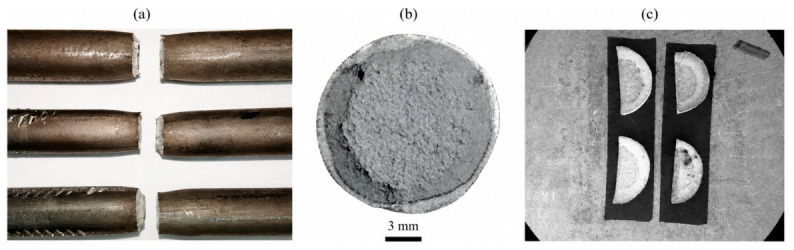
Representative images of fracture-surface specimen preparation and observation: (**a**) fractured 316L/SWRH82B clad wire-rod specimens; (**b**) fracture end face for SEM observation; and (**c**) sectioning and mounting of the fracture end face for subsequent cross-sectional specimen preparation.

**Figure 13:**
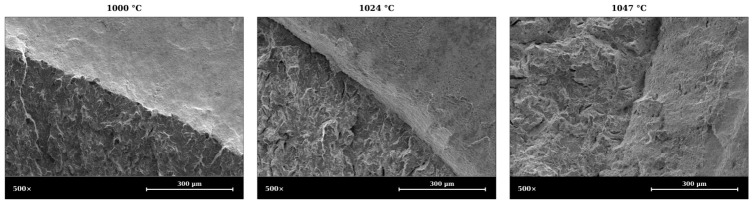
SEM fracture morphologies of the 316L/SWRH82B clad wire rods after tensile testing. Ductile fracture features, including dimples and tearing ridges, are observed without obvious interfacial delamination.

Further SEM examination was performed on the tensile fracture surfaces. The specimen preparation procedure and the schematic illustration of the SEM observation plane are shown in Figure 12, and the corresponding SEM fracture morphologies are presented in Figure 13.

Consistent with the fracture-location records of all 45 tensile specimens, the SEM fractographic results further show that the tensile fracture occurred on the SWRH82B carbon-steel side for all three temperature groups, with fracture surfaces mainly characterized by dimples, tearing ridges, and microvoid coalescence, and no macroscopic peeling, cracking, or brittle delamination along the 316L/SWRH82B interface was observed. This suggests that, within the present industrial hot-rolling window, the fracture mechanism did not fundamentally change from ductile fracture to interfacial brittle failure with increasing initial rolling temperature; instead, effective metallurgical bonding was established in all specimens, and the interfacial fracture resistance exceeded that of the local instability region on the carbon-steel side. Nevertheless, distinct morphological differences were still observed among the three conditions: the 1000 °C specimen showed a relatively clear contrast between locally flat regions and rough dimpled areas, indicating nonuniform plastic deformation and microvoid coalescence; the 1024 °C specimen exhibited more continuous, finer, and more uniformly distributed dimples and tearing features, without obvious layered delamination or large-scale local instability. By contrast, the 1047 °C specimen, although still dominated by ductile fracture, displayed more pronounced surface undulation, tearing ridges, and locally coarse microvoid coalescence, reflecting enhanced local morphological heterogeneity. These SEM differences do not indicate a fundamental change in fracture mode, but rather reflect the evolution of the near-interface microstructural gradient, decarburization-induced softening, and local stress redistribution with increasing initial rolling temperature.

Considering the decarburized layer, carburization-affected zone, elemental distribution and tensile statistics together, the tensile failure behavior can be mainly associated with near-interface microstructural softening and local stress redistribution on the SWRH82B side. With increasing initial rolling temperature, carbon migration from the SWRH82B core to the 316L cladding promoted carbon depletion near the interface, resulting in thickening of the decarburized layer on the carbon-steel side. Meanwhile, local carbon enrichment and possible Cr–C interaction on the 316L side produced an asymmetric near-interface microstructural gradient.

Among the three conditions, the 1024 °C specimen showed the lowest tensile-strength scatter and the highest reduction in area, indicating a better balance between strength stability and plastic deformation capacity. In contrast, although the 1047 °C specimen exhibited the highest average tensile strength, it also showed intensified decarburization, larger cladding-thickness fluctuation and increased mechanical scatter. These results suggest that excessive initial rolling temperature may aggravate local deformation heterogeneity. It should be emphasized that the present tensile and fractographic observations do not directly quantify the intrinsic interfacial bonding strength, because dedicated interfacial shear, peel, bend or ring-compression tests were not performed. Therefore, the results only demonstrate that the 316L/SWRH82B interface was not the preferential macroscopic fracture path under the present axial tensile-loading condition.

### 3.5. Unified Mechanism of Temperature–Diffusion–Microstructure–Geometry–Fracture

**Figure 14:**
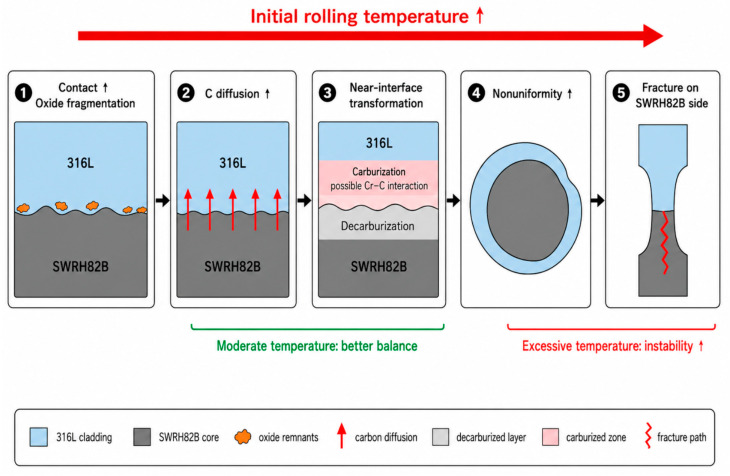
Schematic illustration of the effect of initial rolling temperature on interfacial contact, oxide fragmentation, carbon diffusion, decarburization, carburization-affected zone formation, cladding nonuniformity and tensile fracture path of the 316L/SWRH82B clad wire rod. The upward arrows indicate an increase or enhancement of the corresponding factors with increasing initial rolling temperature.

As schematically summarized in Figure 14, the initial rolling temperature affects the 316L/SWRH82B clad wire rod through a coupled “temperature–interface–diffusion–geometry–fracture” mechanism. With increasing initial rolling temperature, the real interfacial contact area increases, oxide remnants are more effectively fragmented and dispersed, and carbon diffusion from the SWRH82B core toward the 316L cladding is enhanced. These effects promote metallurgical bonding, but they also intensify near-interface reaction–diffusion. As a result, a decarburized layer forms on the SWRH82B side, while a carburization-affected zone and possible Cr–C interaction develop on the 316L side. When the temperature is excessive, the enhanced carbon migration, near-interface microstructural gradient and increased flowability of the 316L cladding jointly aggravate cladding-thickness nonuniformity and local deformation heterogeneity. Consequently, the macroscopic tensile fracture occurs on the SWRH82B side rather than along the 316L/SWRH82B interface, indicating that the fracture-controlling region shifts from the bonded interface to the carbon-steel side affected by diffusion-induced softening and geometrical instability.

Therefore, the preferred initial rolling temperature should be determined by a balance between interfacial bonding, reaction–diffusion moderation, cladding geometrical stability and mechanical consistency, rather than by the highest average tensile strength alone. Under the specific boundary conditions of the present study, namely the 316L/SWRH82B material pair, the composite billet geometry of a 316L tube with an outer diameter of 116 mm and an inner diameter of 106 mm combined with a 104 mm SWRH82B core, and the full-scale 22-pass industrial hot-rolling line, from the investigated initial rolling temperature range of 1000–1047 °C, the finish rolling temperature of 900 ± 20 °C, the coil-laying temperature of 850 ± 20 °C and Stelmor controlled cooling, 1024 °C is identified as the preferred condition among the tested temperatures. This optimum should not be regarded as a universally applicable value, because the present work is limited to as-hot-rolled clad wire rods. Further cold drawing, fatigue and corrosion tests are still required to evaluate drawability and final service performance for bridge cable wire applications.

## 4. Conclusions

This study examines industrially hot-rolled 316L/SWRH82B stainless-steel/high-carbon-steel clad wire rods as precursors for bridge cable wires and clarifies the coupled effects of initial rolling temperature on interfacial evolution, carbon diffusion, cladding stability and tensile fracture behavior. The main conclusions are as follows:

(1) At initial rolling temperatures of 1000, 1024 and 1047 °C, all clad wire rods formed a continuous 316L outer cladding without obvious macroscopic interfacial opening defects. All 45 tensile specimens were fractured in a ductile manner on the SWRH82B side, with no macroscopic peeling or brittle delamination along the 316L/SWRH82B interface, indicating that the interface was not the preferential macroscopic fracture path under the present tensile-loading condition.
(2) Increasing the initial rolling temperature enhanced interfacial reaction–diffusion. The decarburized-layer thickness on the SWRH82B side increased from 7.42 ± 1.28 µm to 11.31 ± 1.74 µm and 18.15 ± 1.76 µm as the temperature increased from 1000 °C to 1024 °C and 1047 °C, respectively. Correspondingly, the carburization-affected-zone thickness on the 316L side increased from 48.36 ± 2.73 µm to 63.04 ± 3.06 µm and 68.73 ± 3.65 µm, respectively, indicating asymmetric interfacial reaction–diffusion.
(3) EPMA results show that the 316L/SWRH82B interface is a reaction–diffusion transition zone involving carbon migration, possible Cr–C interactions, local O/Mn/Si enrichment and possible oxide- or inclusion-related remnants. This transition zone contributes to metallurgical continuity but also introduces near-interface microstructural and property gradients.
(4) The average tensile strengths of the 1000, 1024 and 1047 °C specimens were 1120.07, 1146.27 and 1152.28 MPa, with standard deviations of 14.83, 4.55 and 13.34 MPa, respectively. Although the 1047 °C condition produced the highest average strength, it also caused intensified decarburization, poorer cladding-thickness uniformity and increased mechanical scatter. Therefore, 1024 °C is identified as the preferred condition under the present industrial hot-rolling conditions, rather than a universal optimum.

These findings provide a basis for subsequent studies on cold drawing and corrosion performance of 316L/SWRH82B clad wire rods for bridge cable wire applications. Future work should include drawing trials, evaluation of cladding integrity after drawing, direct interfacial bond testing, and corrosion and fatigue performance assessments.

## Figures and Tables

**Table 1:** Nominal chemical compositions of 316L stainless steel and SWRH82B high-carbon steel according to relevant standards (wt.%).

Material	C	Si	Mn	Cr	Ni	Mo	P	S	Fe
316L	≤0.030	≤0.75	≤2.00	16.00–18.00	10.00–14.00	2.00–3.00	≤0.045	≤0.030	Bal.
SWRH82B	0.79–0.86	0.15–0.35	0.60–0.90	-	-	-	≤0.030	≤0.030	Bal.

**Table 2:** Circumferential cladding-thickness uniformity of the clad wire rods at different initial rolling temperatures.

Initial Rolling Temperature	Maximum Thickness/μm	Minimum Thickness/μm	Mean Thickness/μm	Standard Deviation/μm	Thickness Difference/μm	Coefficient of Variation/%	Tmax/Tmin
1000 °C	976.58	235.39	603.84	161.54	741.19	26.75	4.15
1024 °C	1035.02	297.21	655.67	148.80	737.81	22.69	3.48
1047 °C	1068.26	275.24	623.91	190.65	793.02	30.56	3.88

**Table 3:** Statistical thickness results of the decarburized layer at different initial rolling temperatures (*n* denotes the number of specimens statistically analyzed under each condition).

Initial Rolling Temperature/°C	*n*	Mean ± SD/µm	95% CI/µm	Min–Max/µm
1000	6	7.42 ± 1.28	6.08–8.76	5.29–9.45
1024	6	11.31 ± 1.74	9.48–13.14	7.93–14.85
1047	6	18.15 ± 1.76	16.30–20.00	14.65–21.37

**Table 4:** Statistical thickness results of the carburized layer at different initial rolling temperatures (*n* denotes the number of specimens statistically analyzed under each condition).

Initial Rolling Temperature/°C	*n*	Mean ± SD/µm	95% CI/µm	Min–Max/µm
1000	6	48.36 ± 2.73	45.49–51.23	42.16–54.93
1024	6	63.04 ± 3.06	59.83–66.25	55.45–68.90
1047	6	68.73 ± 3.65	64.90–72.56	59.95–77.40

**Table 5:** Tensile strength and reduction-in-area results of clad wire rods at different initial rolling temperatures.

Initial Rolling Temperature/°C	Average Tensile Strength/MPa	Standard Deviation/MPa	Average Reduction in Area %
1000	1120.07	14.83	36.1
1024	1146.27	4.55	38.9
1047	1152.28	13.34	36.9

## Data Availability

The original contributions presented in this study are included in the article. Further inquiries can be directed to the corresponding author.
